# RELIGION AND TRAJECTORIES OF COGNITIVE FUNCTION AMONG WHITE, BLACK, AND HISPANIC OLDER ADULTS

**DOI:** 10.1093/geroni/igac059.2083

**Published:** 2022-12-20

**Authors:** Madison Sauerteig-Rolston, Lisa Barnes, Patricia Thomas, Kenneth Ferraro

**Affiliations:** Purdue University, West Lafayette, Indiana, United States; Rush Alzheimer’s Disease Center, Rush University Medical Center, Chicago, Illinois, United States; Purdue University--West Lafayette, West Lafayette, Indiana, United States; Purdue University, West Lafayette, Indiana, United States

## Abstract

Most prior research on the relationship between religious involvement and cognition among older adults is based on cross-sectional data and yields inconsistent results. We use longitudinal data from 14,161 older adults in the Health and Retirement study (HRS) to investigate whether religious involvement, measured by attendance, integration, and religiosity (i.e., beliefs, meanings, and values) is associated with trajectories of cognitive function from 2006 to 2016 among a diverse sample of respondents. We find that religiosity is associated with lower levels of cognition at baseline among White adults (b=-0.12, p < 0.001), but higher levels of cognition among Black adults (b=0.18, p < 0.05). In addition, growth curve analysis reveals that religious attendance is associated with higher cognition over time for Hispanic respondents (b=0.07, p < 0.001). Religious involvement is associated with later-life cognition, but this relationship differs for White, Black, and Hispanic older adults.

